# Ruptured phyllodes tumors of the breast: a comprehensive case series and literature review on clinical challenges

**DOI:** 10.1186/s12957-025-03841-y

**Published:** 2025-06-20

**Authors:** Chularat Duangkaew, Areewan Somwangprasert, Kirati Watcharachan, Phanchaporn Wongmaneerung, Chagkrit Ditsatham

**Affiliations:** 1 Division of Head, Neck, and Breast Surgery, Department of Surgery, Faculty of Medicine, Chiang Mai University, 110 Intavaroros Road, Amphoe Muang, Chiang Mai, 50200 Thailand; 2 Clinical Surgical Research Center, Chiang Mai University, Chiang Mai, Thailand

**Keywords:** Ruptured phyllodes tumor, Giant phyllodes tumor, Malignant phyllodes tumor, Phyllodes tumor

## Abstract

Phyllodes tumors are rare fibroepithelial breast neoplasms, predominantly affecting women aged 35 to 55. While most phyllodes tumors are manageable, those exceeding 10 cm, termed giant phyllodes tumors, can lead to rare complications such as breast rupture due to rapid growth and pressure necrosis. This study presents three cases of ruptured phyllodes tumors treated at Chiang Mai University Hospital, along with a review of previously reported cases, highlighting the associated clinical challenges and treatment strategies. Surgical intervention, typically through wide local excision, is the standard treatment. However, in ruptured cases, which often present as extensive masses involving the entire breast, surgical management is more complex and may necessitate a mastectomy. Given the limited number of reported cases and our own findings, the benefit of adjuvant radiotherapy following mastectomy remains unclear. The low recurrence rate among patients who did not receive radiotherapy raises questions about its necessity.

## Introduction

Phyllodes tumors are rare fibroepithelial breast neoplasms, accounting for 0.3–0.9% of all breast tumors worldwide [1]. They can manifest at various ages but are most common between the ages of 35 and 55. The median age at diagnosis is 40, although they can also affect adolescents and the elderly [2, 3]. The tumor was first described in 1838 and initially termed Cystosarcoma Phyllodes due to its histological pattern of “leaf-like” projections into cystic spaces and sarcomatous stroma. The term was later changed to phyllodes tumor to reflect its malignant potential based on pathologic features [3, 4]. The World Health Organization (WHO) classifies phyllodes tumors into benign, borderline, and malignant subtypes, based on criteria including stromal cellularity, stromal atypia, stromal overgrowth, mitotic count, and tumor margins. Surgery with adequate margins remains the primary treatment modality for phyllodes tumors.

Most phyllodes tumors measure 3–4 cm in size [3, 5], and almost 20% of them grow larger than 10 cm in diameter [6], at which point they are referred to as giant phyllodes tumors [5, 7–10], making them more challenging to treat. It is important to note that the rapid growth of a large phyllodes tumor over a short period can cause pressure necrosis of the overlying skin, leading to a ‘rupture’ of the breast [11]. This is an incredibly rare condition, with very few cases have been reported in the literature.

Our study presents the first case series that details the clinical presentation, diagnosis, treatment, and outcomes of three patients with ruptured phyllodes tumors from our institution. In addition, we provide a focused review of previously published case reports, offering a broader perspective on the clinical challenges associated with this rare condition.

## Case presentation

### Case 1

A 56-year-old female, who had previously undergone excisions of left breast masses in 2002 and 2005 with unknown pathological outcomes, presented to the northern regional hospital in 2011. She exhibited a rapidly enlarging 10 cm mass in the central region of her left breast, classified as BIRADS IVb via mammography. Differential diagnoses considered were phyllodes tumor and giant fibroadenoma. Core needle biopsy findings included ductal epithelium and stroma, suggestive of a phyllodes tumor. Despite the recommendation for a left simple mastectomy, the patient declined the surgery and was subsequently lost to follow-up.

**Fig. 1:**
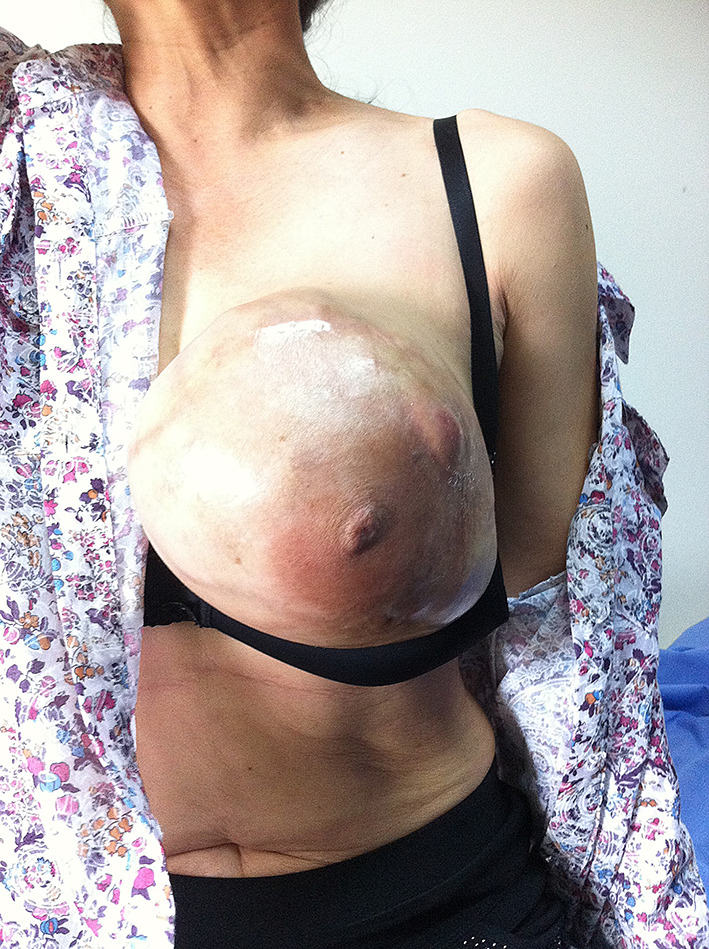
Large phyllodes tumor covering the entire left breast before rupture

**Fig. 2:**
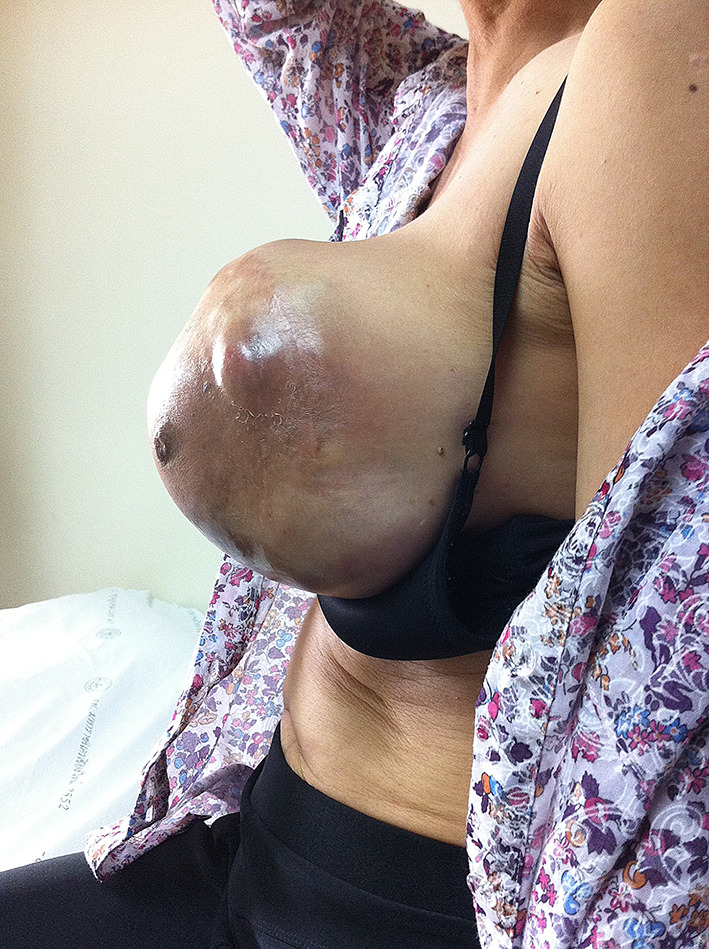
Large phyllodes tumor, lateral view

**Fig. 3:**
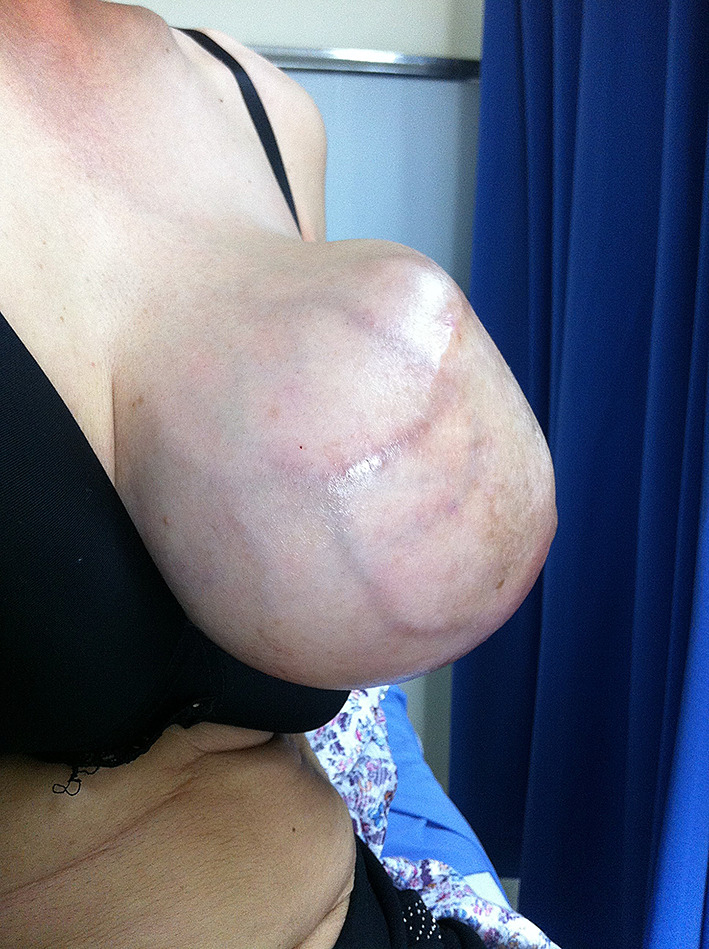
Large phyllodes tumor, medial view

**Fig. 4:**
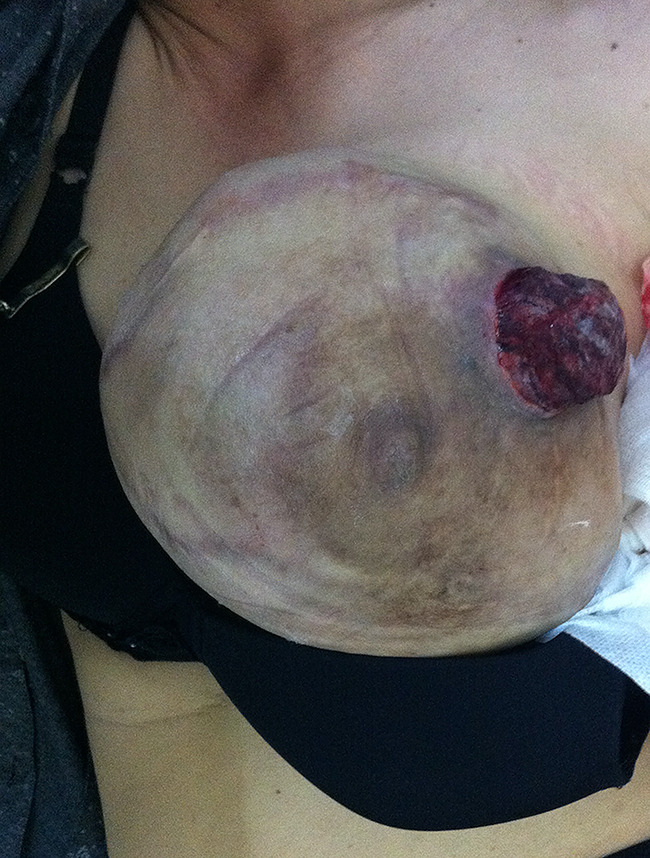
Large phyllodes tumor rupturing through the skin at the upper outer quadrant of the left breast

The patient returned to the regional hospital two months later due to rapid growth of the previously untreated mass. Subsequently, she was referred to the Division of Head, Neck, and Breast Surgery at Chiang Mai University Hospital in April 2014. Initial examination showed a 17 × 15 cm tumor enveloping the entire left breast, as illustrated in Figs. 1, 2 and 3 A left simple mastectomy was planned for the following month; however, ten days before the scheduled surgery, the tumor ruptured through the skin, as detailed in Fig. 4. Antibiotic therapy was initiated.

**Fig. 5:**
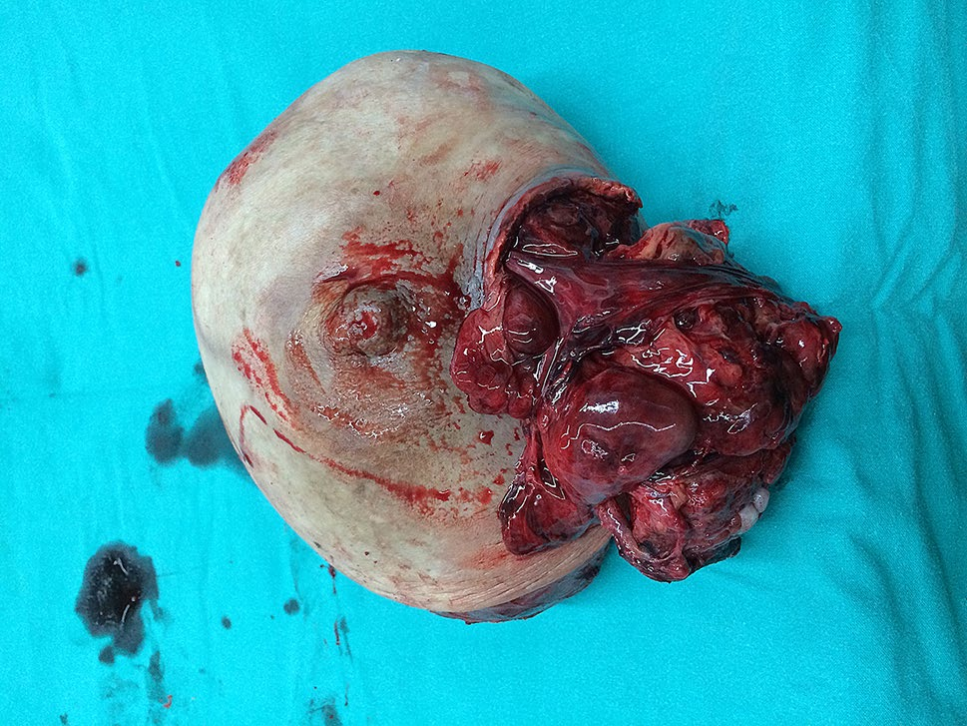
Mastectomy specimen from the left breast showing a ruptured phyllodes tumor

**Fig. 6:**
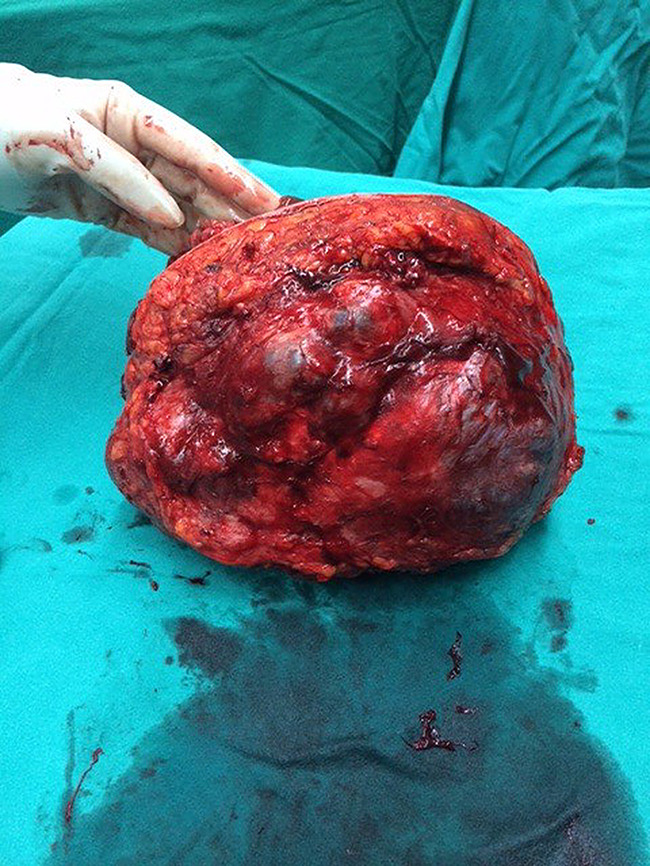
Posterior view of the left mastectomy specimen

A left simple mastectomy was conducted, achieving primary skin closure without the need for reconstructive procedures. The tumor was confined and did not invade adjacent tissues. The resected specimen measured 19 × 18 × 9 cm. Notably, the skin exhibited pressure necrosis extending 5 cm in the upper outer quadrant, with multiple firm nodular masses protruding through the ulceration, as illustrated in Figs. 5 and 6. Pathological examination confirmed a malignant phyllodes tumor encompassing nearly the entire specimen, with the deep margin focally approached by the tumor.

**Fig. 7:**
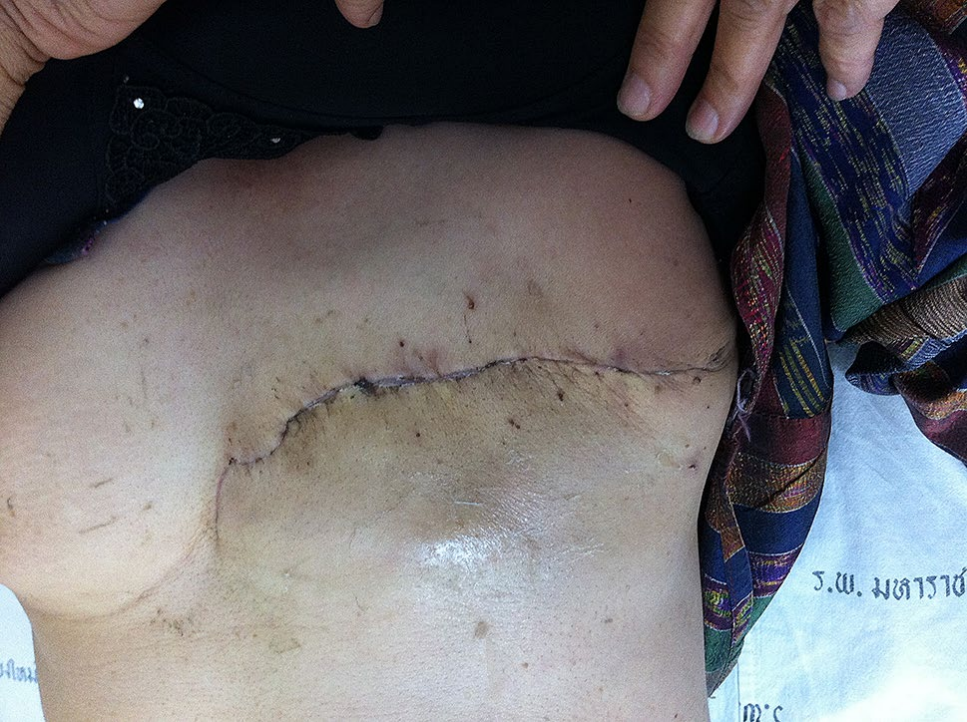
Surgical wound at two weeks post-operation

Subsequently, she received post-mastectomy radiation therapy, as determined during the Breast Planning Conference, where a multidisciplinary team of breast surgeons, medical oncologists, and radiation therapists collaborates. The postoperative mastectomy wound is shown in Fig. 7. Over the subsequent ten years, no recurrence of the tumor has been reported.

### Case 2

A 47-year-old premenopausal woman without underlying medical conditions presented to a provincial hospital due to a rapidly enlarging mass in her right breast. Previously, she underwent a surgery for a benign phyllodes tumor with clear margins at the same hospital in August 2020. Despite the clear margins, the tumor recurred, potentially due to her non-adherence to the recommended follow-up schedule. Eight months before her latest admission, she noticed the mass reappearing, which gradually increased in size and began causing occasional pain. A month before her latest hospital visit, the mass had significantly enlarged, accompanied by serous discharge from the affected area.

**Fig. 8:**
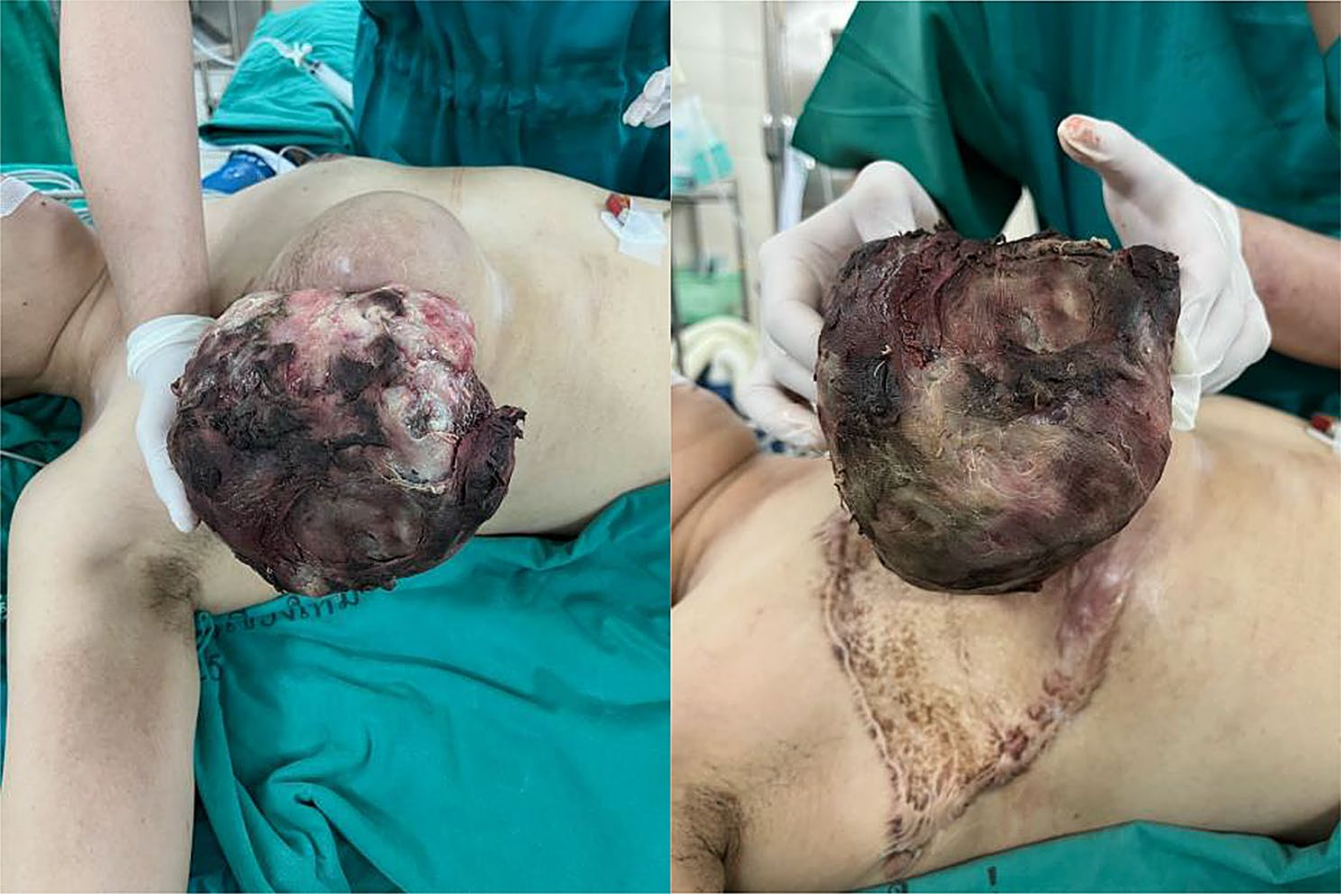
Ruptured phyllodes tumor in a lateral view

**Fig. 9:**
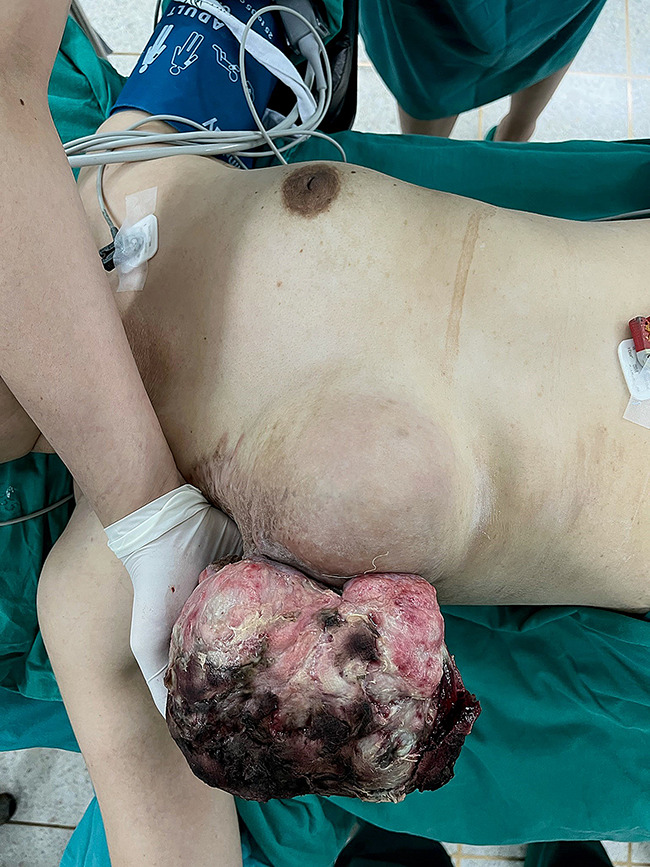
Phyllodes tumor in an anteroposterior view

On physical examination, a mass lesion was observed, occupying the entire right chest wall, accompanied by an ulcerative lesion that bled upon palpation, as illustrated in Figs. 8 and 9. There was no detectable enlargement of the axillary lymph nodes.

A core needle biopsy of the right chest wall mass was performed on September 1, 2022. The biopsy findings suggested a recurrent benign phyllodes tumor, characterized by mildly increased stromal cellularity and atypia, with a mitotic rate of 0–1 per 10 high-power fields. These results supported the clinical diagnosis of a recurrent phyllodes tumor and led to the patient’s admission for surgical management. A wide local excision with delayed reconstruction was scheduled for November 10, 2022.

**Fig. 10:**
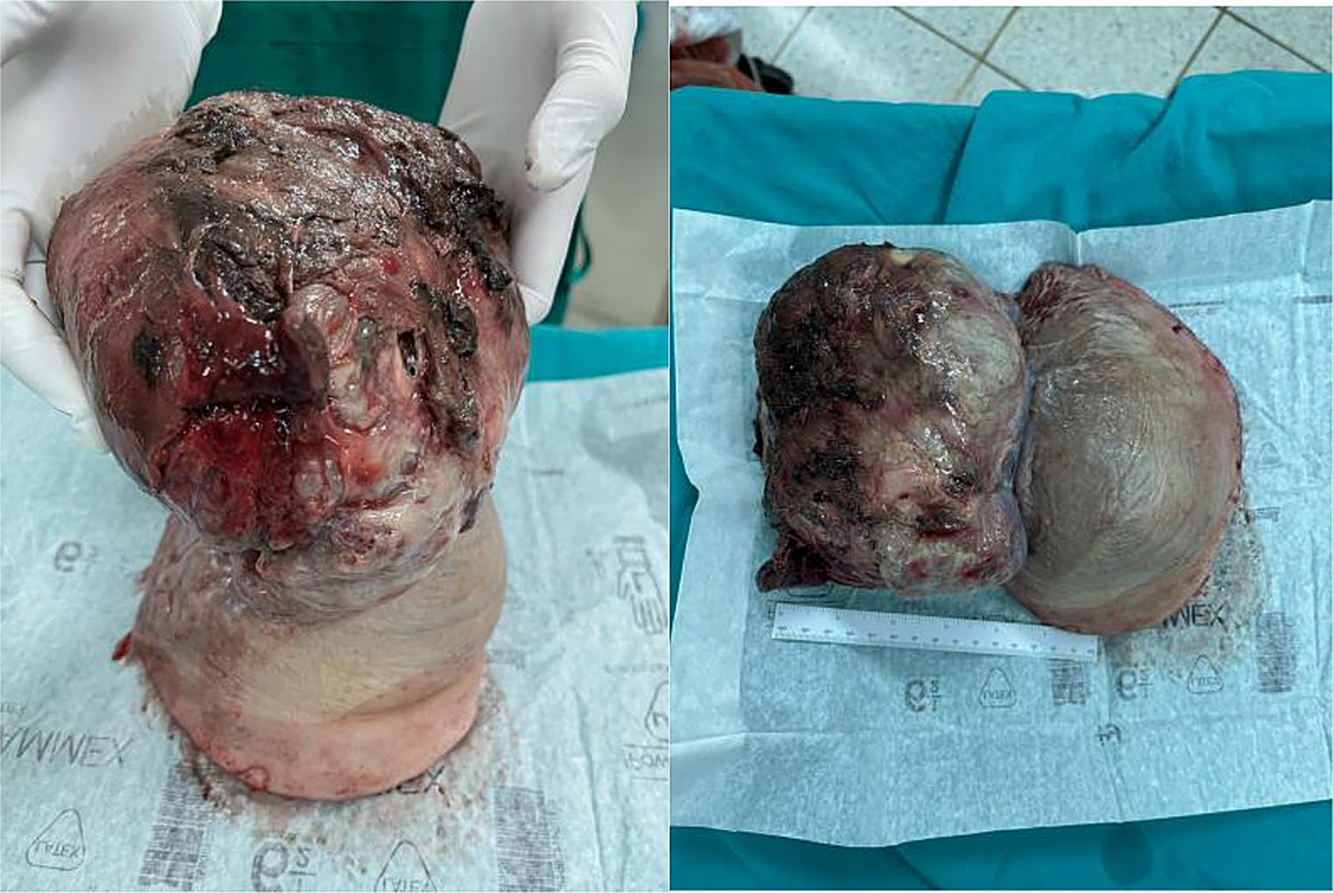
The wide excision specimen of recurrent phyllodes tumor

During the surgical procedure, a phyllodes tumor measuring 15 × 15 cm and weighing approximately 2.5 kg was excised. The tumor was surrounded by an old surgical scar, with clear margins greater than 1 cm, as shown in Fig. 10.

Post-surgical pathology classified the tumor as a borderline phyllodes tumor, measuring 16 cm at its greatest dimension. The pathology report highlighted moderate stromal cellularity with overlapping nuclei and atypia, as well as stromal overgrowth. It documented a mitotic rate of 5 mitoses per 10 high-power fields, and noted the presence of tumor necrosis. The tumor presented a well-defined border with a pushing margin and lacked malignant heterologous elements. A skin ulcer, presumably caused by the tumor’s pressure, was also observed. All surgical margins were clear, with the nearest margin being the deep margin, which had 1 mm of free intervening stroma. Subsequently, the patient underwent a split-thickness skin graft on November 28, 2022. Following the Breast Planning Conference’s decision, the patient received radiation therapy. There has been no recurrence one year and nine months post-surgery.

### Case 3

**Fig. 11:**
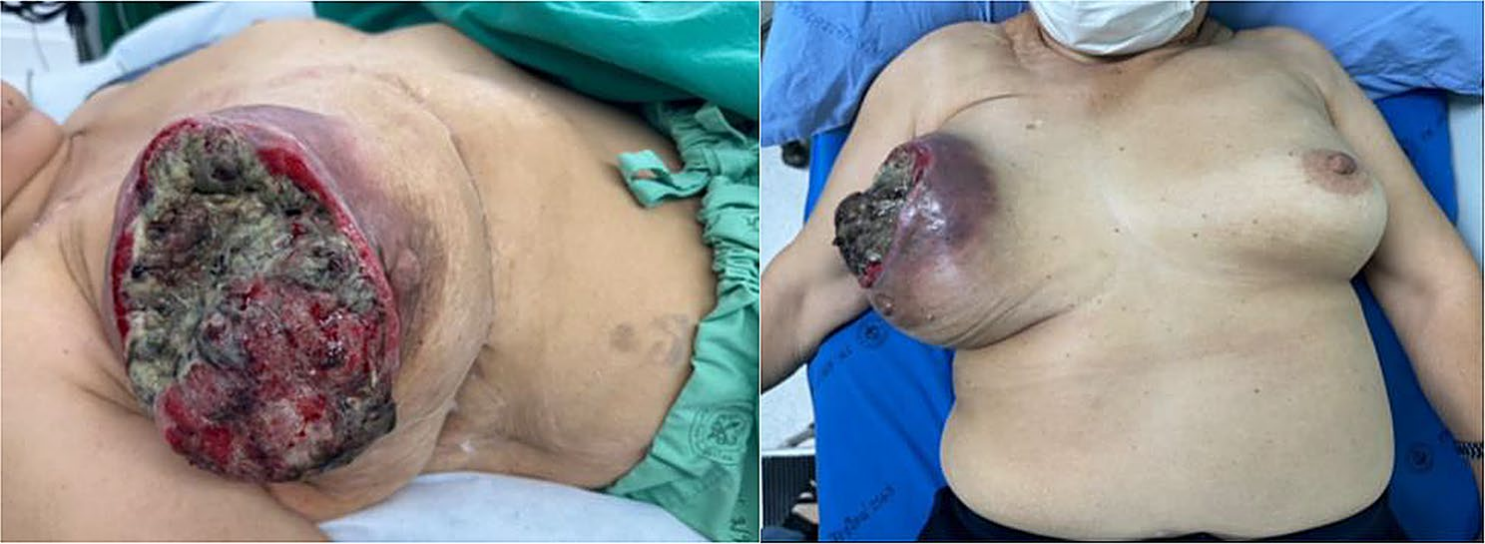
Tumor rupture through the skin in the right breast and the presence of necrotic tissue in the affected quadrant. This visual illustrates the severe progression and aggressive nature of the tumor

A 71-year-old postmenopausal woman with a medical history of hypertension, dyslipidemia, and diabetes, who presented with a rapidly growing mass in her right breast, observed over the past month. The mass was notable for its skin ulceration and occasional bleeding. Physical examination revealed a large, irregular, and nodular mass in the right breast, characterized by ulceration and bleeding, as illustrated in Fig. 11. There was no evidence of axillary lymphadenopathy, and the left breast was unremarkable.

**Fig. 12:**
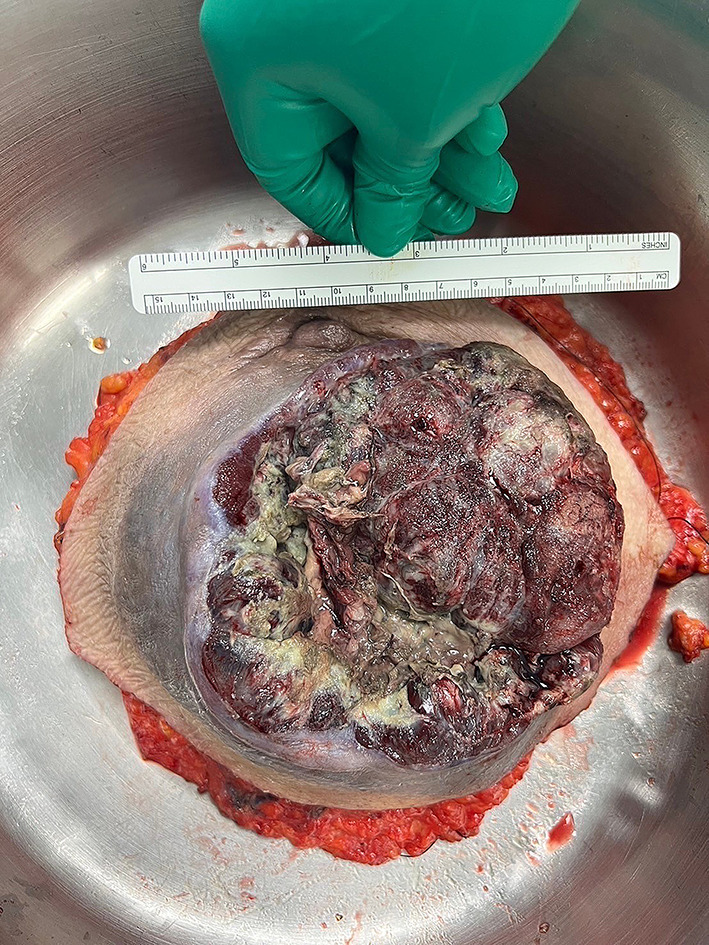
The right mastectomy specimen featuring the ruptured phyllodes tumor. The specimen highlights the extensive damage and invasive growth pattern typical of this aggressive tumor type

A core needle biopsy identified a spindle cell tumor lacking an epithelial component and exhibiting areas of tumor necrosis. The differential diagnosis for this presentation includes phyllodes tumor and spindle cell carcinoma.

Subsequently, the patient underwent an emergency right simple mastectomy due to significant bleeding. The excised tumor measured 13.5 × 13 × 8.5 cm and displayed extensive skin ulceration spanning 12.5 × 12 cm, as illustrated in Fig. 12. Pathological analysis confirmed the tumor as a malignant phyllodes tumor, characterized by a high mitotic rate of 20 mitoses per 10 high-power fields, and margins were clear with a 5 mm clearance at both the anterior and deep margins.

**Fig. 13:**
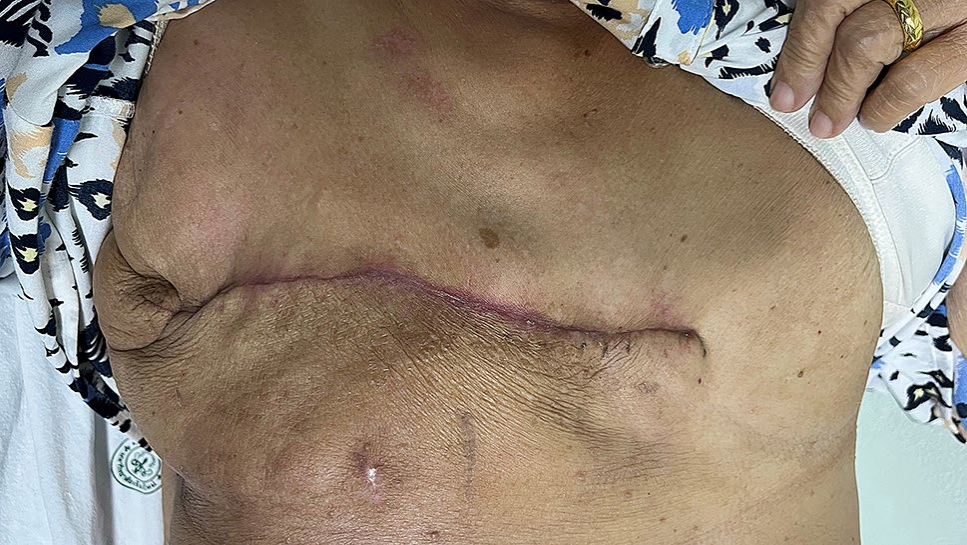
The postoperative mastectomy wound two weeks after surgery

The patient was discharged in good condition on the sixth postoperative day and began receiving post-mastectomy radiation therapy, as determined during the Breast Planning Conference, which was completed in August 2024. The postoperative mastectomy wound is shown in Fig. 13.

## Discussion

Ruptured phyllodes tumors are an uncommon clinical scenario, typically because they seldom grow beyond 10 cm in diameter. However, the rapid enlargement of a large phyllodes tumor within a short period can lead to pressure necrosis of the skin covering it, ultimately causing the breast to rupture [11]. The standard treatment for phyllodes tumors remains surgical intervention, with wide local excision and an adequate margin of at least 1.0 cm being the preferred approach. For borderline or malignant phyllodes tumors, achieving wide surgical margins of at least 1 cm is crucial, as narrower margins are associated with local recurrence rates comparable to those observed with positive margins [12]. In cases of ruptured phyllodes tumors, which often present as extensive masses encompassing the entire breast and causing skin ulceration, achieving these margins can be challenging, frequently necessitating a mastectomy.

Adjuvant systemic treatment is rarely utilized, a randomized controlled trial demonstrated that adjuvant chemotherapy with doxorubicin and dacarbazine did not significantly impact survival in patients with malignant phyllodes tumors [13]. The role of adjuvant radiation therapy remains controversial. A meta-analysis indicated that radiotherapy is effective in controlling local disease and preventing metastasis [14]. A recent study has demonstrated that radiation therapy significantly enhances local recurrence-free survival following margin-negative wide local excision in patients with borderline and malignant phyllodes tumors. However, those who underwent mastectomy did not derive the same benefit from adjuvant irradiation, and it did not improve overall survival [15].

While phyllodes tumors are uncommon, ruptured phyllodes tumors are exceedingly rare, resulting in ongoing debate regarding the treatment strategies for these cases. A literature review identified few studies on ruptured phyllodes tumors, each documenting a single case across benign, borderline, and malignant categories, with malignant phyllodes tumors being most common. Our study is the first case series to detail the clinical presentation, diagnosis, treatment strategies, and outcomes of three patients with ruptured phyllodes tumors, admitted to the Division of Head, Neck, and Breast Surgery at Chiang Mai University Hospital.

Our literature review, summarized in Table 1, reveals that most ruptured phyllodes tumors were malignant and exceeded 9 cm in size, leading to mastectomy in eight of the nine documented cases. Although one patient did not achieve negative margins after mastectomy, no tumor recurrence was observed, and adjuvant radiotherapy was not administered [16]. This observation aligns with a previous study suggesting that patients with borderline or malignant phyllodes tumors treated with mastectomy did not demonstrate improved local recurrence-free survival from adjuvant irradiation compared to those undergoing breast-wide local excision with negative margins [15]. Regarding overall survival, a retrospective study indicated that radiotherapy did not impact long-term survival in patients with malignant phyllodes tumors of the breast who underwent neither wide local excision nor mastectomy [17]. One patient with a ruptured malignant phyllodes tumor experienced local recurrence six months after mastectomy with clear margins but without adjuvant radiotherapy. She subsequently underwent wide excision of a chest wall nodule for the recurrent tumor. Approximately one year later, she developed distant metastasis and organ failure. This is the only case in our literature review in which tumor recurrence was reported. Data on surgical margins in most of the reviewed case reports of ruptured phyllodes tumors were unclear, and the longest follow-up reported was only up to four years. This limitation may not adequately reflect long-term outcomes, such as local recurrence.

**Table 1:** The summarized data of case reports regarding ruptured phyllodes tumors and this study case series

Author and publication	Age	Operation	Subtype	Mass size (cm.)	Nearest Margin, by pathological result (cm.)	RT	Follow-up duration	Follow-up status
**Previous case report**								
Wijeyaratne, 2010 [18]	48	Elective simple mastectomy	Borderline	Un-known	not mentioned	no	4 years	No recurrence
Nabi et al., 2013 [19]	32	Elective wide local excision	Benign	9.5	not mentioned	no	9 months	No recurrence
Ditsatham et al., 2016 [16]	58	Elective simple mastectomy	Malignant	14.8	lateral and deep margins approached	not mentioned	4 months	No recurrence
Meher et al., 2016 [20]	60	Emergency simple mastectomy (due to sepsis)	Malignant	27	not mentioned	no	10 months	No recurrence
Bruce NR, 2017 [11]	60	Elective simple mastectomy	Malignant	9.5	> 2.0	Offered but was denied by patient	Unknown	No recurrence
Yang M, 2022 [21]	70	Emergency simple mastectomy (due to bleeding)	Borderline with foci of malignant phyllode	21	Clear, not mentioned	not mentioned	Unknown	unknown
Matsushima et al., 2024 [22]	51	Emergency simple mastectomy (due to bleeding)	Malignant	10.5	Clear, not mentioned	no	2 years and 10 months	No recurrence
Tong et al., 2024 [10]	50	Elective simple mastectomy	Malignant	20	Clear, not mentioned	no	1 years and 5 months	recurrence at 6 months after 1st surgery
Dong et al., 2024 [23]	48	Elective MRM (ALND was performed due to suspicious metastatic LN	Malignant No LN metastasis	22	not mentioned	no	9 months	No recurrence
**This Study**								
Case 1, 2014	56	Elective simple mastectomy	Malignant	19	deep margin focally approached	Yes	10 years and 2 months	No recurrence
Case 2, 2022	47	Elective wide excision	borderline (rupture at the recurrent period)	16	0.1 (deep margin)	Yes	1year and 9 months	No recurrence
Case 3, 2024	71	Emergency simple mastectomy (due to bleeding)	Malignant	13.5	0.5 (anterior and deep margin)	Yes	1 month	No recurrence

In our case series, three patients diagnosed with ruptured phyllodes tumors underwent wide excision and mastectomy, all of whom subsequently received adjuvant radiotherapy. Despite ongoing debates regarding the efficacy of postmastectomy radiotherapy for borderline and malignant phyllodes tumors [15, 17], our multidisciplinary team opted for adjuvant RT. This decision was influenced by the malignant characteristics of the tumors, the presence of positive margins, and insufficient resection margins—factors independently associated with increased risk of tumor recurrence, as substantiated by systematic reviews and meta-analyses [2]. Notably, our follow-up, which extended up to ten years, showed no recurrence. However, our treatment approach—particularly the use of radiotherapy after mastectomy—differed from previously reported cases, in which most patients did not receive radiotherapy and one recurrence was observed among the nine cases.

Based on data from the literature review and our case series, mastectomy appears to be a suitable treatment option for large ruptured phyllodes tumors. None of the cases in this series received adjuvant systemic therapy. Despite not achieving negative margins, low recurrence was observed in these cases, irrespective of whether the patients received adjuvant radiotherapy. While the role of adjuvant radiotherapy in improving local disease control remains uncertain, its benefit appears limited, particularly given the low recurrence rate observed among patients who did not receive it.

## Conclusion

Phyllodes tumors are rare fibroepithelial tumors of the breast, and ruptured cases are exceptionally uncommon. Although most ruptured cases are malignant, benign and borderline subtypes may also present. Mastectomy often becomes the primary treatment option, particularly when achieving negative margins through wide excision proves infeasible. Based on the limited number of reported cases and our own series, the role of adjuvant radiotherapy after mastectomy remains uncertain. Notably, the low recurrence rate in patients who did not receive radiotherapy suggests its limited benefit, warranting further studies and long-term follow-up.

## Data Availability

All data supporting this study are contained within the paper and its Supplementary Information. Images of patients are included in Figures 1 through 7, 8 through 10, and 11 through 13. A summary of patient data is provided in Table 1.

## Abbreviations

ALND: Axillary lymph node dissection
cm: Centimeter
LN: Lymph node
MRM: Modified radical mastectomy
RT: Radiotherapy
